# GENDER-BASED VIOLENCE IN HOMICIDE–SUICIDE EVENTS: A CALL FOR ADHERENCE TO THE CDC DEFINITION FOR “MERCY KILLING”

**DOI:** 10.1093/geroni/igad104.1838

**Published:** 2023-12-21

**Authors:** Sonia Salari, Carrie Sillito, Emma Videla

**Affiliations:** University of Utah, Salt Lake City, Utah, United States; University of Utah, Salt Lake City, Utah, United States; University of Utah, Salt Lake City, Utah, United States

## Abstract

Intimate partner homicide suicide (IPHS) events are overwhelmingly male perpetrated (90%). Gender-based violence (GBV) prevention requires ‘best practices’ for media reporting. Consider the following: In a patient room of a busy hospital, an overwhelmed man (71) suicided after firing shots at his wife (70) who had survived a stroke. The gunfire caused trauma among witnesses. Media reports used the term ‘mercy killing,’ but critical ingredients were missing. The CDC’s NVDRS (2022) defines ‘mercy killing’ as “…Killed, at the victim’s request…to end…pain or distress…Do not assume…murder/suicide by a sick, elderly couple is mercy killing.” Evidence of a clear wish to die, is needed “due to a hopeless or terminal condition.” Our IPHS, news surveillance study was updated 1999-2022 and used content analysis to identify GBV rationalizations, based upon 2219 events and over 5000 fatalities across young (n=987), middle aged (n=699), and older 60+ (n=533) categories. Only the 60+ group had claims of ‘mercy killing,’ mostly without adherence to the CDC definition. Older men who are suicidal may also harm an unsuspecting partner. For women, end of life decision-making is lost in ‘safe spaces,’ like homes or care facilities, which is made worse by rationalized excuses. Women are rarely suicidal and use passive methods (i.e., drugs). When women care for an ailing partner, they don’t commit IPHS. Older men are more suicidal and utilize more lethal firearm methods (similar to the IPHS patterns). Media best practices involve valuing persons with disabilities, publicizing services and recognizing violence is a public health problem.

